# Anti-Inflammatory Effects of Lipids Extracted from *Arctoscopus japonicus* Eggs on LPS-Stimulated RAW264.7 Cells

**DOI:** 10.3390/md17100580

**Published:** 2019-10-11

**Authors:** Weerawan Rod-in, Chaiwat Monmai, Sang-min Lee, Seok-Kyu Jung, SangGuan You, Woo Jung Park

**Affiliations:** 1 Department of Marine Food Science and Technology, Gangneung-Wonju National University, Gangneung, Gangwon 25457, Koreabbuayy@gmail.com (C.M.); umyousg@gwnu.ac.kr (S.Y.); 2 Department of Marine Biotechnology, Gangneung-Wonju National University, Gangneung, Gangwon 25457, Korea; 3 Department of Horticulture, Daegu Catholic University, Gyeongsan, Gyeongbuk 38430, Korea; seokkyujung@gmail.com

**Keywords:** *Arctoscopus japonicus*, egg, lipid, anti-inflammation, macrophage, NF-κB, and MAPK

## Abstract

*Arctoscopus japonicus* is a cold-water marine fish. The present study investigated the fatty acid composition of *A. japonicus* egg lipids and their anti-inflammatory effects on LPS-stimulated RAW246.7 macrophages. The results showed that *A. japonicus* egg lipids contained primarily polyunsaturated fatty acids (52.9% of the total fatty acid content; mostly eicosapentaenoic acid [EPA, 21.2 ± 0.5%] and docosahexaenoic acid [DHA, 25.9 ± 0.1%]), followed by monounsaturated fatty acids and saturated fatty acids (23.7% and 23.4%, respectively). *A. japonicus* egg lipids significantly decreased nitric oxide (NO) production and suppressed the expression of immune-associated genes such as *iNOS*, *COX-2*, *IL-1β*, *IL-6*, and *TNF-α* LPS-stimulated RAW246.7 macrophages in dose-dependent manner. *A. japonicus* egg lipids also reduced the phosphorylation levels of NF-κB p-65, p38, ERK1/2, and JNK, key components of the NF-κB and MAPK pathways, suggesting that the lipid-induced anti-inflammatory activity is related to these signaling pathways. These results indicate that the lipids extracted from *A. japonicus* eggs have potential biofunctions and might be useful for regulating inflammation in macrophages.

## 1. Introduction

Lipids, more specifically fatty acids, are key components of fish eggs, as they are the source of metabolic energy used for swimming, growth, and reproduction, and the fatty acid proportions in the cell membranes of fish, especially marine fish, are very high [1,2]. The major lipids in fish eggs are composed of saturated fatty acids (SFAs) and polyunsaturated fatty acids (PUFAs), such as eicosapentaenoic acid (EPA, 20:5n-3) and docosahexaenoic acid (DHA, 22:6n-3), and cholesterol [3]. Because of their various bioactivities, lipids from fish eggs have been proposed as nutrients for human health [4]. Lipid mediators derived from PUFAs have been shown to have beneficial effects on inflammatory diseases, such as Alzheimer’s disease [5,6,7], cardiovascular diseases [8,9], asthma [10], inflammatory bowel disease [11], and cancer [12]. They are also involved in fetal development [9], play crucial roles in inflammatory regulation, and contribute to overall health [13,14]. DHA has been shown to reduce interleukin-1β (IL-1β) and tumor necrosis factor (TNF)-α levels in LPS-stimulated peripheral blood mononuclear cells (PBMCs) [15], and linoleic acid (LA; 18:2n-6), α-linolenic acid (ALA, 18:3n-3), and DHA inhibited *IL-1β*, *IL-6*, and *TNF-α* gene expression in human THP-1 macrophages [16].

LPS stimulation of macrophages induces inflammation by activating the nuclear factor-κB (NF-κB) and mitogen-activated protein kinase (MAPK) signaling pathways [17]. NF-κB activity is modulated by phosphorylation and regulatory proteins such as p-65 and IκBα, and upon their degradation, NF-κB translocate to the nucleus where it functions as a transcription factor to regulate inflammation [18]. The MAPK signaling pathway, which includes extracellular signal-regulated kinase (ERK), c-Jun N-terminal kinase/stress-activated protein kinase (JNK), and p38, is involved in various cellular functions, such as cell proliferation, differentiation, and survival [19]. This signaling pathway regulates the expression of various inflammatory mediators, including nitric oxide (NO), inducible nitric oxide synthase (iNOS), and cyclooxygenase (COX)-2, and pro-inflammatory cytokines, such as IL-1β, and IL-6, and TNF-α [20]. Therefore, both the NF-κB and MAPK signaling pathways are primary targets for regulating inflammatory cytokine expression and inflammation-related processes.

The anti-inflammatory activities of lipids derived from marine sources, such as *Katsuwonus pelamis* [21], *Perna canaliculus* [22,23], *Virgularia gustaviana* [24], *Mytilus coruscus* [25], and *Gracilaria* sp. [26], have been studied. *Arctoscopus japonicus*, a popular commercial fish that is widely distributed in the northwestern Pacific Ocean, including the east coast of Korea [27,28], has been reported to contain functional peptides (in the meat and eggs) with antioxidant [29,30] and anti-inflammatory biological activities [31,32]. Although *A. japonicus* has also been shown to possess high lipid contents with biofunctional fatty acids, especially EPA and DHA, few studies have explored the lipids extracted from *A. japonicus* eggs and their anti-inflammatory effects on immune cells [33].

Therefore, the present study analyzed the fatty acid composition of lipids extracted from *A. japonicus* eggs and their anti-inflammatory effects on the immune system using LPS-stimulated RAW264.7 cells.

## 2. Results

### 2.1. Fatty Acid Analysis of A. japonicus Lipids

**Figure 1:**
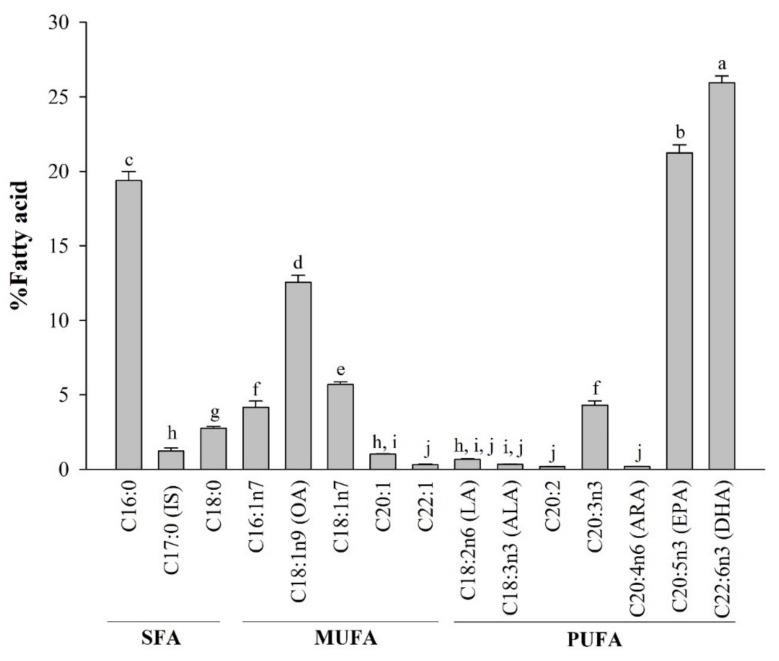
Fatty acid composition of lipids extracted from *A. japonicus* eggs. Data are the mean ± SD (*n* = 5). Lowercase letters (a–j) indicate significant differences (*p* < 0.05) between the amounts of total fatty acid from *A. japonicus* lipids (where, a > b > c > d > e > f > g > h > i > j). SFA, saturated fatty acid; MUFA, monounsaturated fatty acid; PUFA, polyunsaturated fatty acid.

The fatty acid composition of lipids extracted from *A. japonicus* eggs is shown in Figure 1. The fatty acids were first analyzed according to type, i.e., SFA, monounsaturated fatty acids (MUFA), and PUFA. The lipids were mostly composed of PUFAs (52.9%), followed by MUFAs (23.7%) and SFAs (23.4%). Further analysis showed that *A. japonicus* egg lipids contained 19.4 ± 0.6% palmitic acid (C16:0), 2.6 ± 0.1% oleic acid (C18:0), 21.2 ± 0.5% eicosapentaenoic acid (DHA, C20:5n-3), and 25.9 ± 0.5% docosahexaenoic acid (DHA, C22:6n-3).

### 2.2. Cytotoxicity of A. japonicus Egg Lipids

**Figure 2:**
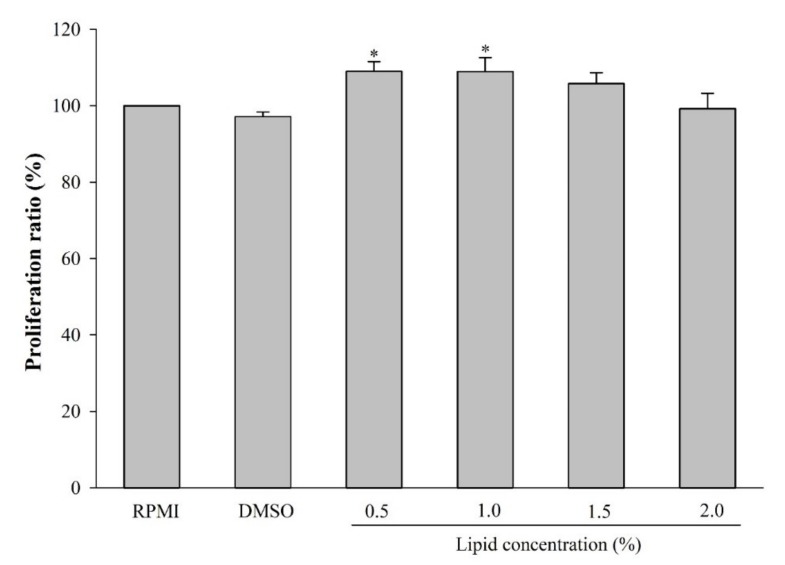
Effect of lipid extracts from *A. japonicus* eggs on the proliferation of RAW264.7 cells. Data are the mean ± SD (*n = 3*). Asterisks indicate a significant difference (*p* < 0.05) compared to cells incubated with RPMI (set at 100%).

To examine the potential toxicity of *A. japonicus* egg lipids on RAW264.7 cells, the cells were incubated with different concentrations of *A. japonicus* egg lipids (0%, 0.5%, 1.0%, 1.5%, and 2.0%), and cell viability was assessed. As shown in Figure 2, *A. japonicus* egg lipids did not decrease cell viability, but certain concentrations moderately stimulated the proliferation of RAW264.7 cells.

### 2.3. Effects of A. japonicus Egg Lipids on NO Production

**Figure 3:**
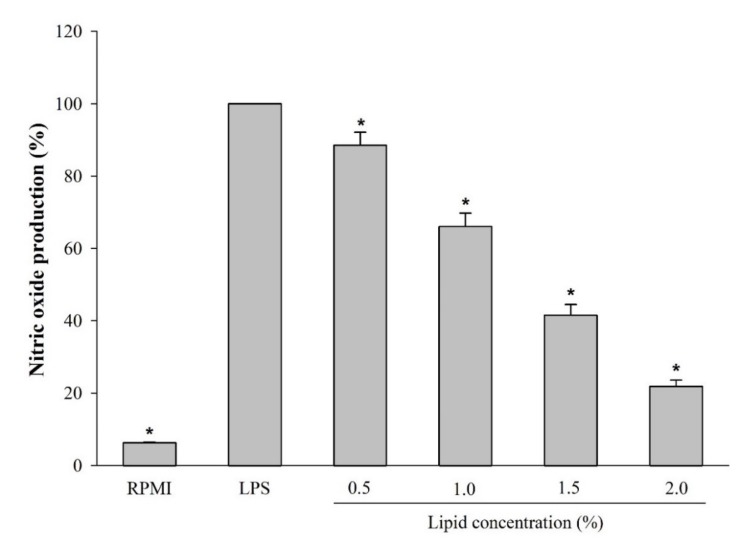
Effect of lipids extracted from *A. japonicus* eggs on NO production in LPS-stimulated RAW264.7 cells. Data are the mean ± SD (*n* = 3). Asterisks indicate a significant difference (*p* < 0.05) compared to LPS.

To evaluate the effect of *A. japonicus* lipids on immune regulation, NO production by RAW264.7 cells was assessed in the presence of extracted *A. japonicus* egg lipids. Figure 3 shows that NO production was significantly reduced in the presence of 0.5–2.0% lipids in a concentration-dependent manner.

### 2.4. Anti-Inflammatory Effect of A. japonicus Egg Lipids Mediated by Modulation of Immune-Associated Gene Expression

**Figure 4:**
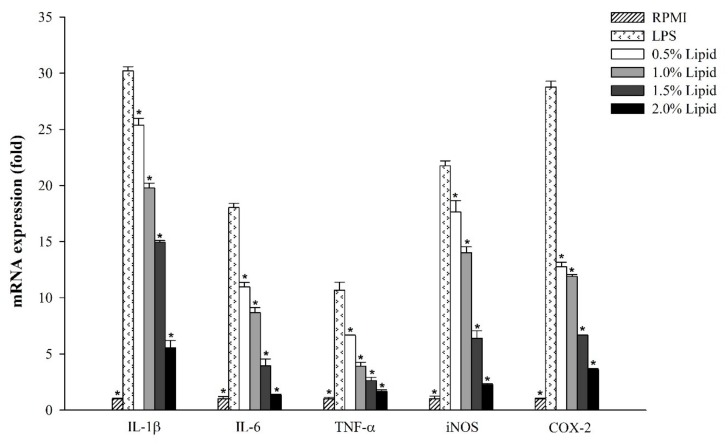
Effects of lipids extracted from *A. japonicus* eggs on the expression levels of immune-associated genes in LPS-stimulated RAW264.7 cells. Data are the mean ± SD fold difference compared to unstimulated cells (*n = 3*). Asterisks indicate significant differences (*p* < 0.05) versus LPS alone.

The effects of lipids extracted from *A. japonicus* eggs on the expression levels of immune-associated genes in LPS-stimulated RAW264.7 cells were examined by quantitative real-time PCR. The results showed that *A. japonicus* lipids decreased the expression levels of most tested genes and significantly reduced the expression levels of the inflammatory mediators *iNOS* and *COX-2* as well as the pro-inflammatory cytokines *IL-1β*, *IL-6*, and *TNF-α* in an *A. japonicus* egg lipid concentration-dependent manner (Figure 4).

### 2.5. Anti-Inflammatory Effects of A. japonicus Egg Lipids Involve the NF-ĸB and MAPK Signaling Pathways

**Figure 5:**
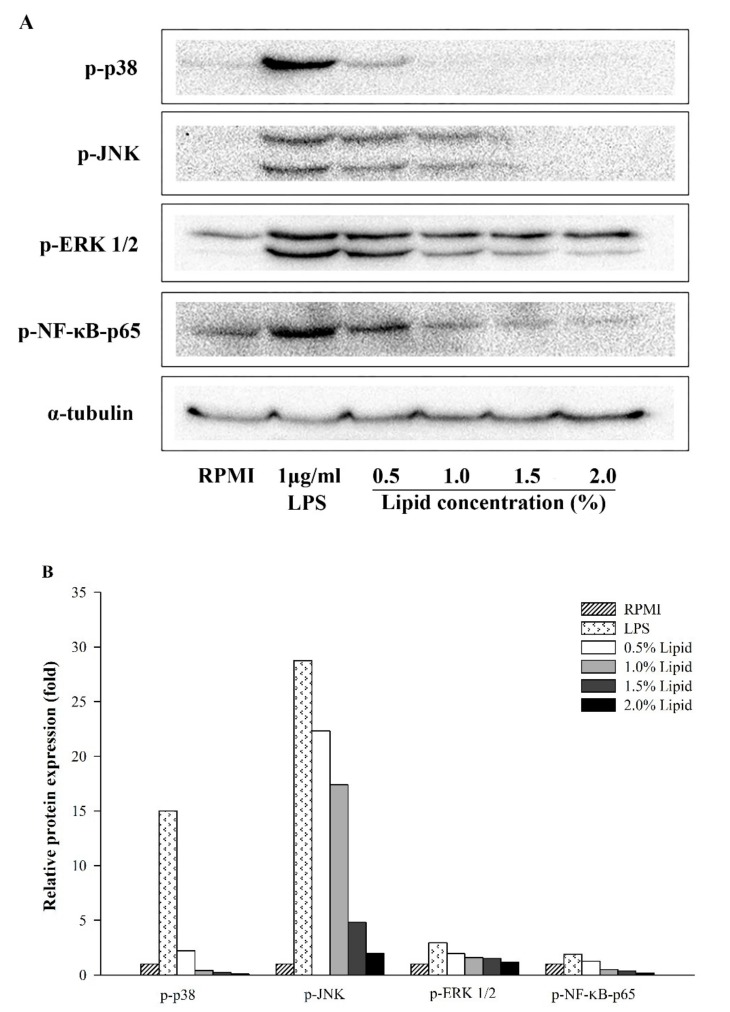
Effect of lipids extracted from *A. japonicus* eggs on the protein levels in the NF-κB and MAPK pathways in LPS-stimulated RAW264.7 cells as determined by western blotting. (A) Western blot; (B) quantification of relative band intensity.

To investigate whether the lipid extracts from *A. japonicus* eggs influence immune-associated signaling, the pathways involving NF-κB and MAPKs were evaluated by western blotting. As shown Figure 5, *A. japonicus* egg lipids decreased the phosphorylation levels of the NF-κB p65 subunit in a concentration-dependent manner, when compared to that in the control. The levels of phosphorylated ERK, JNK, and p38, which are the biomarkers of the MAPK signaling pathway, were also reduced in lipid-treated RAW264.7 cells.

## 3. Discussion

*A. japonicus*, a cold-water fish found in the northwestern Pacific Ocean, the East Sea of Korea, and the Northern Sea of Japan, possesses lipids containing high levels of PUFAs, especially EPA and DHA, which are commonly found in fish and marine foods [34] and are useful in the pharmaceutical and food industries for their beneficial effects on human health [35]. However, no studies have examined the lipids from *A. japonicus* eggs and their anti-inflammatory effects on murine RAW264.7 cells.

The total fatty acid composition of the lipids extracted from *A. japonicus* eggs was analyzed by GC-FID (Figure 1), which showed that the predominant fatty acids were DHA, (22:6n-3), EPA (20:5n-3), and ALA (18:3n-3). In addition, the major SFAs and MUFAs were palmitic acid (16:0) and oleic acid (OA, 18:1n-9), respectively. Similar to *A. japonicus* egg lipids, lipids extracted from Pollock eggs were shown to include fatty acids with high levels of palmitic acid and oleic acid as well as DHA and EPA [36], whereas tuna eggs contained much higher levels of DHA (26.19%) than those of EPA (3.80–4.62%) when compared with *A. japonicus* egg lipids [37].

Palmitic acid is an essential source of metabolic energy in fish during growth and egg formation in female fish [38]. Oleic acid, a major MUFA, plays a key role in energy metabolism during fish spawning [38]. Likewise, *A. japonicus* eggs may require these SFAs for energy metabolism during embryonic development. Our results showed that the dominant fatty acids were PUFAs, which likely fulfill the nutritional requirements of the immune system during embryonic development [39]. These results suggested that the lipids extracted from *A. japonicus* eggs, especially the high EHA and DHA contents, may be involved in inflammation regulation in RAW264.7 cells.

Macrophages play a key role in the regulation of acute and chronic inflammation by removing antigens and increasing of NO production [40]. Prostaglandin E2 (PGE2), a key inducer of inflammatory symptoms, such as fever, swelling, and pain, was evaluated in activated macrophage cells [41]. Under inflammatory conditions, i.e., LPS stimulation, macrophages were activated to induce the production of inflammatory mediators such as NO and PGE2, which was mediated by inducible nitric oxide synthase (iNOS) and cyclooxygenase (COX)-2, respectively, as well as pro-inflammatory cytokines such as IL-1β, IL-6, and TNF-α [42]. Our results demonstrated that *A. japonicus* egg lipids significantly decreased NO production and the expression of immune-associated genes such as *iNOS*, *IL-1β*, *IL-6*, *TNF-α*, and *COX-2* in LPS-stimulated RAW264.7 cells. These results indicated that *A. japonicus* egg lipids have potential for use as anti-inflammatory regulators.

NF-κB, a well-known transcription factor, modulates the expression of genes involved in the innate and adaptive immune-associated genes such as *iNOS*, *COX-2*, and pro-inflammatory cytokine [18]. Moreover, MAPKs, such as ERK1/2, JNK, and p38, are involved in the expression regulation of these immune-associated genes under inflammatory conditions [20,43]. In addition to its effects on NO production and immune-associated gene expression, *A. japonicus* egg lipids also reduced the phosphorylation of NF-κB p-65 and various MAPKs (p-38, JNK, and ERK), suggesting their involvement in the observed anti-inflammatory activity. JNK signaling may be a major pathway because JNK protein phosphorylation was remarkably reduced by *A. japonicus* egg lipids in LPS-stimulated RAW264.7 cells.

## 4. Materials and Methods 

### 4.1. Preparation of A. japonicus Lipids

*A. japonicus* was obtained from the East Sea near Gangwon province, South Korea, and its eggs were isolated, freeze-dried, and ground for lipid extraction using a modification of the method of Bligh and Dyer [44]. *A. japonicus* egg lipids were prepared by extraction using chloroform and methanol and inert gas evaporation. After dissolving the evaporated sample in dimethyl sulfoxide (DMSO), it was weighed to determine the lipid mass and stored at −20 °C.

### 4.2. Fatty Acid Analysis

The fatty acids were extracted from *A. japonicus* lipids according to the method of Garces and Mancha [45]. Fatty acid methyl esters (FAMEs) were prepared by the modified one-step lipid extraction method [46] to analyze the fatty acid composition. The FAMEs were analyzed by gas chromatography (GC)-flame ionization detection (FID) (Perkin Elmer, Waltham, MA, USA).

### 4.3. Cell Culture

The RAW264.7 cells line was obtained from the Korean Cell Line Bank (Korean Cell Line Research Foundation, Seoul, Korea), and cultured in RPMI-1640 medium supplemented with 10% fetal bovine serum and 1% penicillin/streptomycin at 37 °C in a humidified atmosphere containing 5% CO_2_.

### 4.4. Cell Viability

After a 24-h incubation in 96-well plates, RAW264.7 cells were cultured with five different concentrations of *A. japonicus* lipids (0%, 0.5%, 1.0%, 1.5%, and 2.0%) for another 24 h. After discarding the supernatant, cell proliferation was evaluated by using the EZ-Cytox Cell Viability Assay Kit (Daeil Lab Service, Korea) as described by Kim et al. [47]. The ratio of proliferating cells was calculated according to the following equation:

$$\mathrm{Proliferation}\;\mathrm{ratio}\;\left(\%\right)=\frac{\mathrm{Absorbance}\;\mathrm{of}\;\mathrm{the}\;\mathrm{sample}}{\mathrm{Absorbance}\;\mathrm{of}\;\mathrm{the}\;\mathrm{control}}\;\times \;100$$

### 4.5. Nitric Oxide (NO) Production

RAW264.7 cells were cultured with the different concentrations of *A. japonicas* lipids for 1 h. After incubation, the cells were treated with 1 μg/ml LPS for 24 h. Then, NO production was determined based on nitrite accumulation in the culture medium using Griess reagent (Promega, USA) [48].

### 4.6. RNA Isolation and Real-Time PCR

Total RNA was isolated from RAW264.7 cells using TRI reagent® (Molecular Research Center, Inc., USA), and then reverse transcribed using the High Capacity cDNA Reverse Transcription kit (Thermo Scientific, Waltham, MA, USA), according to the manufacturer’s instructions. The mRNA levels of inflammatory genes were quantified by quantitative real-time PCR using SYBR® Premix Ex Taq™ II (Takara Bio, Inc., Kusatsu, Japan) and a QuantStudio™ 3 FlexReal-Time PCR System (Thermo Scientific, Waltham, MA, USA). The primers used in this analysis are shown in Table 1. The results were quantified using the 2^−ΔΔC^_T_ method [49]. β-Actin was included as a control gene.

### 4.7. Western Blotting

Cultured RAW264.7 cells were lysed using RIPA buffer (Tech & Innovation, Hebei, China) to extract total proteins from the cells, and the protein was quantified with the Pierce™ BCA Protein Assay Kit (Thermo Scientific, Waltham, MA, USA). Total proteins were separated by SDS-polyacrylamide gel electrophoresis (SDS-PAGE) and then transferred to a polyvinylidene fluoride (PVDF) membrane, and an immunoblot assay was carried out as described by Narayanan et al. [50]. To investigate the NF-ĸB and MAPK signaling pathways, related proteins were detected with antibodies against phosphorylated NF-ĸB p65 (Cell Signaling Technology, #3033), p38 (Cell Signaling Technology, #9211), JNK (Cell Signaling Technology, #9251), and ERK1/2 (Cell Signaling Technology, #9101) along with α-tubulin (Abcam, #ab15246) as a control, and goat anti-rabbit IgG (H+L)-HRP (GenDEPOT, SA006-500). The signals were measured using Pierce® ECL Plus Western Blotting Substrate (Thermo Scientific, Waltham, MA, USA). The blot was quantitatively analyzed using a ChemiDoc XRS+ imaging system (Bio-Rad, Hercules, CA, USA) and ImageLab software (version 4.1, Bio-Rad).

### 4.8. Statistical Analysis

SPSS 24.0 software (SPSS, Chicago, IL, USA) was used to analyze the study results. Data are reported as mean ± standard deviation (SD) and were analyzed by one-way analysis of variance (ANOVA) followed by Duncan’s multiple-range test, with statistical significance set at *p* values less than 0.05.

## 5. Conclusions

Our study demonstrated that lipids extracted from *A. japonicus* eggs reduced the LPS-induced expression and levels of inflammatory mediators and pro-inflammatory cytokines by suppressing the NF-κB and MAPK signaling pathways in RAW264.7 macrophages. These results suggest that *A. japonicus* egg lipids have anti-inflammatory properties and are a potential biofunctional, anti-inflammatory, marine lipid material. Further studies are needed to confirm whether total lipids from *A. japonicus* eggs exhibit anti-inflammatory effects in inflammatory disease models.

## Figures and Tables

**Table 1:** Oligonucleotide primers used in this study.

Gene	Accession No.	Primer Sequence (5’ to 3’)
IL-1β	NM_008361.4	Forward: GGGCCTCAAAGGAAAGAATC Reverse: TACCAGTTGGGGAACTCTGC
iNOS	BC062378.1	Forward: TTCCAGAATCCCTGGACAAG Reverse: TGGTCAAACTCTTGGGGTTC
IL-6	NM_031168.2	Forward: AGTTGCCTTCTTGGGACTGA Reverse: CAGAATTGCCATTGCACAAC
COX-2	NM_011198.4	Forward: AGAAGGAAATGGCTGCAGAA Reverse: GCTCGGCTTCCAGTATTGAG
TNF-α	D84199.2	Forward: ATGAGCACAGAAAGCATGATC Reverse: TACAGGCTTGTCACTCGAATT
β-Actin	NM_007393.5	Forward: CCACAGCTGAGAGGGAAATC Reverse: AAGGAAGGCTGGAAAAGAGC
